# Milk miRNAs: simple nutrients or systemic functional regulators?

**DOI:** 10.1186/s12986-016-0101-2

**Published:** 2016-06-21

**Authors:** Bodo C. Melnik, Foteini Kakulas, Donna T. Geddes, Peter E. Hartmann, Swen Malte John, Pedro Carrera-Bastos, Loren Cordain, Gerd Schmitz

**Affiliations:** Department of Dermatology, Environmental Medicine and Health Theory, University of Osnabrück, Osnabrück, Germany; School of Chemistry and Biochemistry, Faculty of Science, The University of Western Australia, Crawley, Australia; Center for Primary Health Care Research, Lund University, Lund, Sweden; Department of Health and Exercise Science, Colorado State University, Fort Collins, USA; Institute of Clinical Chemistry and Laboratory Medicine, University Clinics of Regensburg, Regensburg, Germany

**Keywords:** Endocytosis, Exosomes, Gene regulation, Intestinal uptake, Lactation, Mammary gland, Milk, miRNA, miRNA degradation, Mouse models

## Abstract

Milk is rich in miRNAs that appear to play important roles in the postnatal development of all mammals. Currently, two competing hypotheses exist: the *functional hypothesis*, which proposes that milk miRNAs are transferred to the offspring and exert physiological regulatory functions, and the *nutritional hypothesis*, which suggests that these molecules do not reach the systemic circulation of the milk recipient, but merely provide nutrition without conferring active regulatory signals to the offspring. The functional hypothesis is based on indirect evidence and requires further investigation. The nutritional hypothesis is primarily based on three mouse models, which are inherently problematic: 1) miRNA-375 KO mice, 2) miRNA-200c/141 KO mice, and 3) transgenic mice presenting high levels of miRNA-30b in milk. This article presents circumstantial evidence that these mouse models may all be inappropriate to study the physiological traffic of milk miRNAs to the newborn mammal, and calls for new studies using more relevant mouse models or human milk to address the fate and role of milk miRNAs in the offspring and the adult consumer of cow’s milk.

## Introduction

Milk contains a plethora of miRNAs, molecules that are known to play pivotal roles in the post-transcriptional regulation of gene expression in various organisms [1, 2]. Milk miRNAs are known to remain stable under adverse conditions, including RNase digestion, low pH, high temperature, and freeze/thaw cycles in the case of frozen milk [3–6]. Milk miRNAs are not only present in free form within the liquid part of milk (skim milk), but they are also packaged inside carrier vehicles, which include milk exosomes, milk cells as well as other microvesicles such as the milk fat globules [6]. This packaging of milk miRNAs has been suggested to further mediate their protection after milk ingestion, potentially facilitating their absorption in the suckling young [6]. Indeed, it has been recently demonstrated that populations of milk cells survive in the gastrointestinal tract of suckling mouse pups and enter the systemic circulation through which they are transferred to and integrate in various organs [7, 8]. This, in addition to the previously described exosomal transfer of milk miRNAs [9], may provide an alternative route of protection, absorption and function of milk miRNAs in the young. Further, milk exosomal miRNA profiles change in response to mammary gland infection [2]. Similarly, milk cellular miRNA profiles have been shown to change in response to feeding. Together, the various levels of protection of miRNA within milk, their dynamic nature, and the fact that most milk miRNAs originate from the lactating mammary epithelium [10, 11] provide indirect evidence supporting specific function(s) of these molecules during lactation, for both the mammary gland and the recipient young [6]. However, animal studies conducted so far have reported controversial findings, some being in support and others against the hypothesis of the functional significance of milk-derived miRNAs in the offspring. Here, the unsuitability of the mouse models previously used to examine the migration and potential function of milk miRNA in the offspring is discussed, lessening the current controversy and supporting the transfer via multiple routes and potential function of milk miRNAs in the young. Further studies are urgently required using suitable animal models as well as in humans to fully address the transfer and functional significance of milk-derived miRNAs in the offspring and the adult human consumer of cow’s milk.

**Fig. 1:**
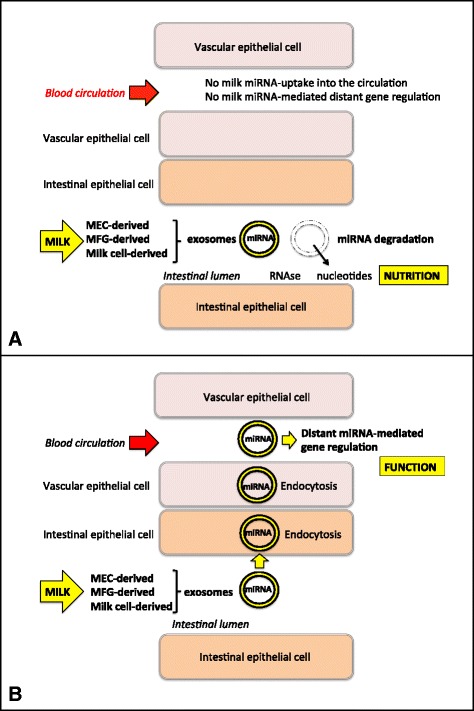
Illustration of the nutritional hypothesis (**a**) and the functional hypothesis (**b**) for the fate of milk-derived miRNAs. The nutritional hypothesis claims that milk miRNAs are degraded in the intestinal lumen and only provide nucleotides that serve local nutritional requirements for intestinal growth or the growth of other organs via the bloodstream. According to the functional hypothesis, milk-derived miRNAs are absorbed into the bloodstream via endocytosis of exosomes, other milk microvesicles or the direct transfer of milk cells, and exert distant gene regulatory functions. MC: Milk cell; MEC: mammary epithelial cell; MFG: Milk fat globule

Currently, two hypotheses exist: the *functional hypothesis*, which accepts the transfer and function of milk miRNAs in the offspring, and the *nutritional hypothesis*, which suggests that these molecules do not reach the systemic circulation of the milk recipient, but merely provide nutrition without conferring active regulatory signals to the offspring (Fig. 1). The functional hypothesis is based on indirect evidence outlined above and requires further investigation. The nutritional hypothesis is based on three problematic mouse models: 1) miRNA-375 KO mice, 2) miRNA-200c/141 KO mice, and 3) transgenic mice presenting high levels of miRNA-30b in milk. We will present circumstantial evidence that these three models may all be inappropriate to study the physiological traffic of milk miRNAs from the mammary gland to mammalian offspring.

### Transgenic mice overexpressing miRNA-30b

miRNA-30b is a critical miRNA involved in the control of lactation [12]. Transgenic mice overexpressing miRNA-30b show a reduction in the size of alveolar lumen, a defect of the lipid droplets and a growth defect of pups fed by transgenic females [12]. The defect in mammary epithelial cell biology caused by overexpression of miRNA-30b may impair cellular traffic and correct assembly of milk exosomes. Laubier et al [13] used this model to examine milk exosomal miRNA traffic in the offspring and found no effect of the elevated miRNA-30b level in the mouse milk on its level in pup tissues. The authors reported that the concentration of miRNA-30b in the milk of transgenic mice was 134 times the concentration in the wild-type control. However, they did not assess whether the extra miRNA-30b in the milk of this model was encapsulated in extracellular vesicles such as exosomes. miRNA encapsulation plays an essential role for mRNA transport and stability [14–17]. The fact that miRNA-30b concentration in the stomach of transgenic pups was only 31 times the concentration in the wild-type pups, i.e. substantially lower than the ratio in milk, is consistent with an extravesicular localization resulting in impaired stability and bioavailability of miRNA-30b from these transgenic mice. This confounder was disregarded by Laubier et al [13] and is an important shortcoming of this study. It is therefore inappropriate to use a pathological lactation model for the study of physiological milk exosome pathways. Notably, the pups of these mice have severe growth defects. It is not unlikely that exosomal components important for endocytotic exosome uptake are missing in this model. Thus, transgenic miRNA-30b overexpressing mice with lactation defects and disturbances of mammary epithelial cell differentiation are not suitable for studying milk exosome traffic under physiological conditions. An aberrant composition of miRNA-30b-containing milk exosomes may explain the observed failure of miRNA-30b intestinal uptake [13]. Furthermore, it has been recently demonstrated that increased levels of miRNA-30b inhibit phagocytosis in myeloid inflammatory cells [18]. Taken together, this model appears to be inappropriate for the study of the complex pathway of maternal-neonatal milk exosome trafficking.

### miRNA-375 and miRNA-200c/141 KO mice

Title et al [19] studied two genetic models of miRNA-375 and miRNA-200c/141 knockout (KO) mice, which received milk from wild-type foster mothers. No convincing evidence was presented of intestinal milk miRNA uptake, but rather rapid intestinal miRNA degradation, leading the authors to conclude that milk miRNAs do not play a gene regulatory role in newborn mammals but may only serve as a nutritional source. Yet, a small increase in the plasma levels of both of these miRNAs was detected in KO pups after nursing, therefore the authors did not exclude the possibility that some miRNA copies may be transferred to the bloodstream, although they did not consider this small transfer to have a biological effect. However, miRNAs have been shown to function even at very low concentrations, of the femtomolar to picomolar range [20–22]. Notably, as has been previously discussed [6], neither of the examined miRNAs (miR-375 and miR-200c) in the study of Title et al [19] was highly expressed in the wild-type mother’s milk of this murine model, whilst both of these miRNAs are known to be involved in the control of endocytosis and/or exocytosis and to modulate epithelial function, which may influence exosome endocytosis and hence their uptake. For instance, modulation of miRNA-375 expression alters voltage-gated Na(+) channel (VGNC) properties and exocytosis in insulin-secreting cells [23]. VGNCs modify the endocytotic membrane activity of human breast and prostate cancer cells [24]. KRas, a target of miRNA-200c, is involved in the control of endocytosis and/or exocytosis [25, 26]. Further, most recent studies have shown that miRNA-375 misses a miRNA sequence motif {(A/U)(C2-4)(A/U)} that is essential for miRNA packaging into exosomes [27]. Thus, the miRNA-375 and miRNA-200c KO mice appear to also be inappropriate models to study milk exosome uptake, which may be critically dependent on physiological miRNA-375 and miRNA-200c signaling involved in endocytotic exosome pathway regulation.

Cells appear to take up microvesicles by a variety of endocytic pathways, including clathrin-dependent endocytosis, and clathrin-independent pathways such as caveolin-mediated uptake, macropinocytosis, phagocytosis, and lipid raft-mediated internalization [28]. Munagala et al. [29] demonstrated transport and bioavailability of fluorophore-labeled bovine milk exosomes in mice. Izumi et al. [30] showed that bovine milk exosomes containing miRNA and RNA are taken up by human macrophages. Wolf et al [9] provided evidence for an intestinal transport of bovine milk exosomes by endocytosis. Kusuma et al [31] reported recently that vascular endothelial cells take up bovine milk exosomes via endocytosis [31]. Moreover, they showed that fluorophore-tagged bovine milk exosomes accumulate in non-intestinal tissues following oral administration in mice [31]. In accordance, Arntz et al [32] demonstrated active uptake of bovine milk exosomes by murine intestinal cells. In 2014, Baier et al [33] reported a dose-dependent increase of miRNA-29b and miRNA-200c in blood serum after cow’s milk consumption in healthy adult human subjects. Interestingly, Aucherbach et al [34] in 2016 could not reproduce these findings when analyzing the samples provided by the laboratory of Baier et al [33] and concluded that there is no evidence for a transfer of bovine milk mRNAs into the circulation of adult humans. However, prolonged sample storage over months and temperature changes during sample transport (loss of dry ice reported by Auerbach et al [34]) may have impaired exosome integrity and thus miRNA recovery, especially in enzymatically highly active peripheral blood mononuclear cells.

In the pig and wallaby, specific milk miRNAs mirrored increased serum levels of lactation-derived miRNAs of the suckling newborns, further supporting an intestinal uptake of milk-derived miRNAs [11, 35]. The majority of milk miRNAs are endogenously synthesized in mammary epithelial cells and these molecules are abundant in human milk, further supporting lactation-specific function(s) [10, 11, 36]. Remarkably, the 14 highly expressed miRNAs of bovine milk fractions are related with target genes associated with organismal development such as hematological, cardiovascular, skeletal, muscular, and immune system development [37] favoring a systemic gene-regulatory role of milk-derived miRNAs [36]. Milk of humans and livestock animals is enriched with immune-related miRNAs [36, 38], which may not only shape the intestinal immune system [39], but may also support the development of thymus-controlled immune regulation [40], both via microvesicle-associated miRNAs and miRNAs contained within milk cells, which have recently been shown to be actively transferred to the thymus of suckling mouse pups (Alsaweed M et al 2016, personal communication).

## Conclusions

Collectively, at present no direct evidence exists that convincingly demonstrates exosomal and other vehicle-mediated uptake of milk miRNAs under physiological conditions, whilst the animal models that have investigated this thus far are considered unsuitable in many respects. However, indirect evidence based on numerous miRNA stability studies, in vitro exosomal trafficking studies, and ex vivo human and animal milk miRNA origin and content studies strongly suggests a function in the recipient offspring [6, 40–44]. In this regard, intestinal permeability has to be considered which is increased during the postnatal period and in inflammatory bowel diseases [45, 46]. Increased intestinal permeability may promote intestinal uptake of miRNA-enriched milk exosomes as well as milk cells. It is critical that a physiological feeding study is conducted with healthy newborn as well as adult animals given radiolabelled milk exosomes that can distinguish between exogenous uptake and endogenous synthesis of these molecules as well as human milk studies to convincingly examine the fate and function of these milk-derived molecules in the recipient offspring and the adult human consumer of pasteurized cow’s milk.

## Abbreviations

KO, knock out; MiRNA, micro ribonucleic acid; VGNA, voltage-gated Na(+) channel
